# IncM Plasmid R1215 Is the Source of Chromosomally Located Regions Containing Multiple Antibiotic Resistance Genes in the Globally Disseminated *Acinetobacter baumannii* GC1 and GC2 Clones

**DOI:** 10.1128/mSphere.00117-16

**Published:** 2016-06-08

**Authors:** Grace A. Blackwell, Mohammad Hamidian, Ruth M. Hall

**Affiliations:** School of Molecular Bioscience, The University of Sydney, Sydney, New South Wales, Australia; Swiss Federal Institute of Technology Lausanne

**Keywords:** *Acinetobacter baumannii*, genomic resistance islands, IS*26*, IncM plasmid

## Abstract

Clear similarities between antibiotic resistance islands in the chromosomes of extensively antibiotic-resistant isolates from the two dominant, globally distributed *Acinetobacter baumannii* clones, GC1 and GC2, suggest a common origin. A close relative of the likely progenitor of both of these regions was found in R1215, a conjugative IncM plasmid from a *Serratia marcescens* strain isolated prior to 1980. The 37.8-kb resistance region in R1215 lies within the *mucB* gene and includes *aacC1*, *aadA1*, *aphA1b*, *bla*_TEM_, *catA1*, *sul1*, and *tetA*(A), genes that confer resistance to gentamicin, streptomycin and spectinomycin, kanamycin and neomycin, ampicillin, chloramphenicol, sulfamethoxazole, and tetracycline, respectively. The backbone of this region is derived from Tn*1721* and is interrupted by a hybrid Tn*2670* (Tn*21*)-Tn*1696*-type transposon, Tn*6020*, and an incomplete Tn*1*. After minor rearrangements, this R1215 resistance island can generate AbGRI2-0*, the predicted earliest form of the IS*26*-bounded AbGRI2-type resistance island of GC2 isolates, and to the multiple antibiotic resistance region (MARR) of AbaR0, the precursor of this region in AbaR-type resistance islands in the GC1 group. A 29.9-kb circle excised by IS*26* has been inserted into the *A. baumannii* chromosome to generate AbGRI2-0*. To create the MARR of AbaR0, a different circular form, again generated by IS*26* from an R1215 resistance region variant, has been opened at a different point by recombination with a copy of the *sul1* gene already present in the AbaR precursor. Recent IncM plasmids related to R1215 have a variant resistance island containing a *bla*_SHV_ gene in the same location.

**IMPORTANCE** Two lineages of extensively antibiotic-resistant *A. baumannii* currently plaguing modern medicine each acquired resistance to all of the original antibiotics (ampicillin, tetracycline, kanamycin, and sulfonamides) by the end of the 1970s and then became resistant to antibiotics from newer families after they were introduced in the 1980s. Here, we show that, in both of the dominant globally disseminated *A. baumannii* clones, a related set of antibiotic resistance genes was acquired together from the same resistance region that had already evolved in an IncM plasmid. In both cases, the action of IS*26* was important in this process, but homologous recombination was also involved. The findings highlight the fact that complex regions carrying several resistance genes can evolve in one location or organism and all or part of the evolved region can then move to other locations and other organisms, conferring resistance to several antibiotics in a single step.

## Abstract (precis)

Two lineages of extensively antibiotic-resistant *A. baumannii* currently plaguing modern medicine each acquired resistance to all of the original antibiotics (ampicillin, tetracycline, kanamycin, and sulfonamides) by the end of the 1970s and then became resistant to antibiotics from newer families after they were introduced in the 1980s. Here, we show that, in both of the dominant globally disseminated *A. baumannii* clones, a related set of antibiotic resistance genes was acquired together from the same resistance region that had already evolved in an IncM plasmid. In both cases, the action of IS*26* was important in this process, but homologous recombination was also involved. The findings highlight the fact that complex regions carrying several resistance genes can evolve in one location or organism and all or part of the evolved region can then move to other locations and other organisms, conferring resistance to several antibiotics in a single step.

## INTRODUCTION

Gram-negative bacteria share a relatively small number of genes that confer resistance to the original antibiotics (such as ampicillin, tetracycline, kanamycin, and sulfonamides). These genes have all been shown to be included within mobile elements that facilitate their spread, namely, compound or class I transposons, class II transposons, or class 1 integrons and their associated gene cassettes. In early resistant isolates, the transposons were generally intact. However, over time more complex resistance regions have emerged as various mobile elements have acted on one another. These regions include several resistance genes either in transposons located within other transposons, such as Tn*2670* (1) or Tn*4* (2). They are frequently found in plasmids that carry horizontal transfer functions, allowing them to spread into multiple strains and species. For example, Tn*2670*, which includes Tn*21*, and Tn*10* were first found in the FIIA plasmid NR1 (or R100) in the late 1950s (reviewed in reference 1), Tn*1696* was found in the IncP plasmid R1033 in 1979 (3, 4), and Tn*1721* was found in pRSD1 in 1979 (5). As further events took place, resistance genes were both added and lost, and rearrangements also occurred, these regions evolved to become increasingly complex arrangements of a series of fragments, each derived from an original transposon. Indeed, as many complete plasmid sequences have become available, evidence has emerged that much of the evolution of large resistance regions occurs within the boundaries of a transposon that is located in a specific position. For example, the class 1 integron in SGI1 genomic islands found in *Salmonella* and *Proteus* chromosomes has many internal configurations (reviewed in reference 6), many A/C plasmids carry a form of the ARI-A resistance island (7), which has a fixed location but varied gene content (reviewed in reference 8), and evolution of a resistance region *in situ* has also been reported for IncHI1 plasmids (9) and IncL/M plasmids (10). The final arrangements can include characteristic specific junctions, such as the exact location of a transposon or insertion sequence (IS), or the junction between regions derived from different mobile elements, which allow larger complex regions with a shared history to be identified.

**FIG 1:**
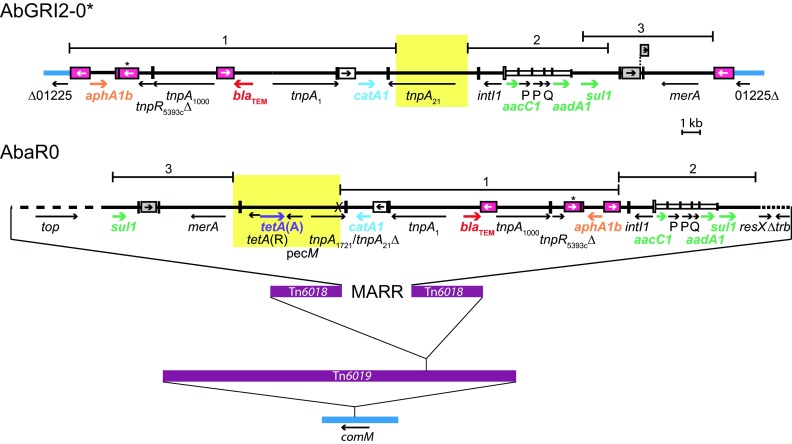
Relationship between AbGRI2-0* and the MARR of AbaR0. Thick blue lines depict adjacent chromosomal sequence portions. The thick black solid and dashed lines indicate sequences within the resistance island of AbGRI2-0* and the MARR of AbaR0. Arrows below diagrams indicate the extent and orientation of some of the genes and open reading frames, with antibiotic resistance genes shown in color IS are boxes and the internal arrow depict the orientation of the *tnp* transposase gene. The boxes highlighted in pink are IS*26*, and an asterisk indicates that the IS*26* is 3 nucleotides different from the standard sequence. White and gray boxes represent IS*1* and IS*6100*, respectively, and IS*6100*Δ is drawn above the main line. Inverted repeats are shown as vertical lines. The lines above indicate regions of identical sequence, while sequences within the yellow boxes are unique to each island. The corresponding locus tag of the gene interrupted by AbGRI2-0* from the genome of A1 (GenBank accession number CP010781) is provided. The thick maroon lines represent the backbone transposons of AbaR0.

*Acinetobacter baumannii* is an important nosocomial pathogen whose treatment is increasingly problematic due to high levels of antibiotic resistance. Both globally disseminated clones, global clones 1 and 2 (GC1 and GC2), contain several antibiotic resistance genes in chromosomally located resistance islands that are made up of transposons and fragments of transposons. GC1 isolates carry a single region designated AbaR (11–16), whereas two resistance islands are found in most GC2 isolates (17–19). The multiple antibiotic resistance region (MARR) in the center of AbaR islands in GC1 isolates and AbGRI2, found in recent GC2 isolates, have been found to share some features (17). Recently, we determined the structure of AbGRI2 of the earlier GC2 reference, RUH134/A320, which was isolated in 1982, and we predicted the ancestral arrangement, AbGRI2-0* (20), which is shown in the top line in Fig. 1. Comparison of AbGRI2-0* to the MARR of AbaR0, the ancestral AbaR island (21), revealed large regions of identical sequence (Fig. 1), suggesting a common origin. However, both resistance islands contain sequence that is not present in the other (highlighted in yellow in Fig. 1), indicating that the source should include both of these segments.

Here, an IncM plasmid, R1215, recovered from a *Serratia marcesens* strain that was isolated in or prior to 1980 and stored as NCTC 50331 in the National Collection of Type Cultures was found to contain resistance genes present in AbGRI2 and AbaR-type resistance regions, and the plasmid sequence was determined. The R1215 resistance island was compared to both the AbaR MARR and AbGRI2.

## RESULTS

### Structure of R1215.

A group of 16 *Escherichia coli* strains containing plasmids of known incompatibility type obtained from the United Kingdom National Collection of Type Cultures (NCTC) for use as references for PCR-based replicon typing was screened for resistance phenotype and, using PCR, for resistance and other genes as described elsewhere (22). We noticed that R1215, which confers resistance to ampicillin, chloramphenicol, gentamicin, kanamycin, streptomycin, sulfonamide, and tetracycline, contained all of the genes found in the AbaR islands of multiply antibiotic-resistant isolates belonging to GC1 of *A. baumannii*. Specifically, R1215 contained a class 1 integron with the *sul1* gene in the 3′-conserved segment (3′-CS), the gene cassette array *aacC1*-orfP-orfP-orfQ-*aadA1* (conferring resistance to gentamicin and spectinomycin), and a *merA* gene identical to that found in Tn*1696*. It also included the *bla*_TEM_ ampicillin resistance gene, the *tet*(A) tetracycline resistance determinant, *catA1*, which confers resistance to chloramphenicol, and the *aphA1b* kanamycin and neomycin resistance gene. Using primers designed to amplify the MARR of AbaR (14), some of the characteristic junctions were found to be present in R1215.

As it was a possible source of the *A. baumannii* resistance islands, the plasmid was purified and sequenced. The sequence reads assembled into three contigs, which were linked by PCR, with the final structure confirmed by restriction digestion. R1215 is a 95,855-bp plasmid (GenBank accession number KU315015) that belongs to the IncM1 incompatibility group, as recently defined by Carattoli and coworkers (23). The antibiotic resistance genes were clustered together in a 37,808-bp region that interrupts the *mucB* gene and is surrounded by a 5-bp duplication of the target site (5′-AAATA-3′). The plasmid backbone obtained by removing the resistance region was found to be 99.9% identical to the backbone of several IncM plasmids for which sequences are available in GenBank (the closest relatives are listed in Table 1 and described in more detail below). Like these plasmids, R1215 includes a set of genes required for conjugative transfer. R1215 was shown to conjugate by transferring it into an *E. coli* recipient. All resistances associated with the donor strain were transferred, and the *repM* replicon was shown to be present in the transconjugants.

**TABLE 1:** IncM1 plasmids related to R1215

Plasmid	Plasmidsize (bp)	GenBankID	Species	Location	Yrisolated	Backbonesize (bp)	RI size(bp)	Resistancegenespresent	Reference
R1215	95,856	KU315015	*S. marcescens*		<1980	58,043	37,808	*aphA1b*, *bla*_TEM_, *catA1*, *aacC1*, *aadA1*, *sul1*, *tetA*(A), *merA*	This study
pACM1	89,977	KJ541681	*K. oxytoca*	United States	1995	58,130	31,842	*bla*_SHV-5_, *dfrA1*, *aadA1*^a^, *aacA4*, *aacC1*, *merA*, *tetA*(A)	28
pIGT15	74,839	KP294351	*E. coli*	Poland	2005	47,849^b^	26,985	*bla*_SHV-5_, *aacA4*, *aacC1*, *aadA1*, *sul1*, *merA*, *tetA*(A)	31
pARM26	86,948	KP294350	Uncultured	Poland	2011	58,128	28,815	*bla*_SHV-5_, *aacA4*, *aacC1*, *aadA1*, *sul1*, *merA*, *tetA*(A)	31
p202c	79,502	KM406490	*S. enterica*	Albania	1985	52,483^c^	27,019	*bla*_SHV-5_, *aacA4*, *aacC1*, *aadA1*, *sul1*, *merA*, *tetA*(A)	31

a pACM1 contains two copies of the *aadA1* gene.

b Missing 10.28 kb in the backbone, including *radC*, *rmoA*, and *klcA*.

c There is a 4.35-kb deletion in the backbone left of the RI.

### Structure of the resistance region.

**FIG 2:**
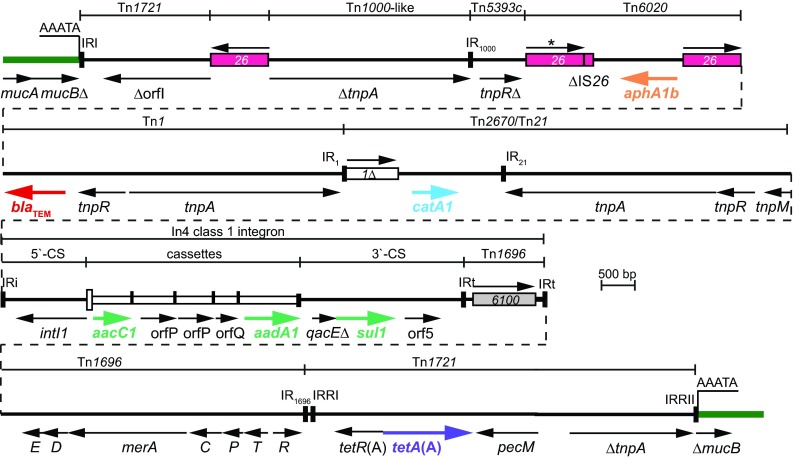
Resistance island in R1215. Thick green lines indicate adjacent plasmid backbone, and black lines represent sequence of the resistance island. The extent and orientation of some of the genes and open reading frames are indicated by the arrows below the line, and antibiotic resistance genes are shown in color. The origin of different DNA segments is shown above. Insertion sequences are shown as boxes with arrows above indicating the orientation of the *tnp* transposase gene and numbers inside indicating the identity of the IS. IS26* differs by 3 nucleotides from the standard sequence of IS*26.* Inverted repeats are shown as vertical lines. The *attI1* site of the class 1 integron is shown as a tall open box, and each narrow box represents a cassette with a vertical bar that indicates the *attC* site. The 5-bp target site duplication flanking the island is underlined.

The resistance region (Fig. 2) is bounded by remnants of Tn*1721* (5), and the *tetA*(A) tetracycline resistance determinant is present at the right-hand end. A transposon with a hybrid Tn*21*-Tn*1696* backbone is found close to IRRI of Tn*1721*, and at the *tnp* end of this transposon the sequence extends into the *catA1*-IS*1* configuration found next to Tn*21* in Tn*2670*. However, the outer end of IS*1* has been truncated by 32 bp due to the insertion of Tn*1*, carrying the *bla*_TEM_ gene, and at the other end the Tn*1* has itself been truncated by IS*26* and lacks the final 129 bp at the *bla*_TEM_ end. The IS*26* forms one end of Tn*6020*, an IS*26*-bounded transposon, which includes the *aphA1b* gene that was originally found in *A. baumannii* (13). The final fragment is composed of one end of a transposon related to Tn*1000* and a small internal fragment of Tn*5393*c, followed by IS*26*; again, this configuration was first seen in *Acinetobacter* (11, 13). The length and identity of the various segments are summarized in Table 2.

**TABLE 2:** Modules in the resistance region in R1215

Regionno.^a^	Length(bp)	Progenitor				Region orgene(s)	Identity(%)
		Name	Length(bp)	GenBankaccession no.^b^	Portionpresent^c^		
1	1,707	Tn*1721*	11,128	AB366441.1^d^	9422–11128	ΔorfI	100
2	820	IS*26*	820	AY123253	Complete	IS*26*	100
3	2,883^e^	Tn*1000*	5,981	X60200	1–2883	*tnpA*	74
4	685	Tn*5393c*	5,470	AF313472	2968–3652	Δ*tnpR*	99.9
5	3,069	Tn*6020*	3,069	FJ172370	1–3069	*aphA1b*	100
6	4,820	Tn*1*	4,949	HM804085	130–4949	*bla*_TEM_, *tnpR*, *tnpA*	99.8
7	6,264	Tn*2670*	22,760	AP000342	38–6301	ΔIS*1*, *catA1*, *tnpA*, *tnpR*, *tnpM*	100
8	731	IS*1*	768	AP000342	38–768	ΔIS*1*	100
9	4,039	Tn*21*	19,672	AF071413	1–4039	*tnpA*, *tnpR*, *tnpM*	100
10	1,371	5′-CS^f^	1,371	U12338	Complete	5′-CS^f^	99.6
11	2,908	Cassettes	2,908	AF453999	1–2908	*aacC1*-orfP-orfP-orfQ-*aadA1*	99.6
12	7,731	Tn*1696*	16,318	U12338	8285–16318	3′-CS^f^-IS*6100*-*mer1696*	99.9
13	5,568	Tn*1721*	11,139	X61367^d^	1–5568	Δ*tnpA*-*tetR*-*tetA*(A)-pecM-Δ*tnpA*	100

a The analysis begins with the first base of the resistance region in R1215 (GenBank accession number KU315015).

b The accession number used was either that for the earliest complete sequence or for the complete and annotated sequence.

c Numbering for an IS includes the transposase gene from left to right; for class II Tn, the *tnpA* gene is on the left.

d X61367 is the original complete GenBank entry for Tn*1721*, but this sequence has several single-base differences and a short deletion which are also not present in other published sequences of this region and might be errors.

e Of the region’s length, 209 bp were 99.6% identical to a Tn*1000*-like element found in GenBank sequence accession number AY598759.

f 5′-CS and 3′-CS are the 5′-conserved segment and 3′-conserved segment of a class 1 integron.

Not only does the R1215 resistance region carry the same cassette array as AbaR0 (21), but also all of the boundaries between the DNA segments derived from different sources are identical to those in AbaR and AbGRI2 islands (indicated in Fig. 4 in reference 20). Furthermore, the two segments that are present only in either AbaR or in AbGRI2 islands (highlighted in Fig. 1) are both present in R1215. These features are consistent with the resistance region of R1215 being the source or closely related to the source of the antibiotic resistance genes in both of the *A. baumannii* resistance islands.

### From R1215 to AbGRI2-0*.

**FIG 3:**
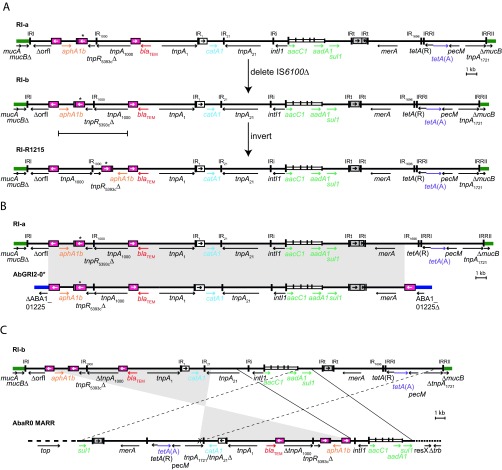
Conversion of the resistance island in R1215 to AbGRI2-0* and the MARR of AbaR0. (A) Predicted precursor RI-a and RI-b of the R1215 resistance island. (B) Relationship between RI-a and AbGRI2-0*. (C) Relationship between RI-b and the MARR of AbaR0. Thick solid green, blue, and black lines represent sequences of the plasmid backbone, chromosome, or the resistance island, respectively. The arrows below indicate the extent and orientation of some of the genes and open reading frames. Antibiotic resistance genes are shown in color. Inverted repeats are shown as vertical lines. Insertion sequences are shown as boxes, and pink, white, and gray boxes represent IS*26*, IS*1*, and IS*6100*, respectively. An asterisk indicates the IS*26* is 3 nucleotides different from the standard sequence. The arrows within the boxes indicate the orientation of the *tnp* transposase gene. The *attI1* site of the class 1 integron is shown as a tall open box, and each narrow box represents a cassette with a vertical bar that indicates the *attC* site. In panels B and C, gray blocks of shading and dashed and solid lines between the rows indicate regions of identical sequence.

To simplify the description of the pathway to AbaR0 and AbGRI2-0*, we invoked precursors RI-a and RI-b for the R1215 resistance region (Fig. 3). In both precursors, the region between the outer copies of IS*26* is inverted relative to the R1215 RI, and this could have occurred via recombination between two inversely oriented copies of IS*26*. In RI-a, the IS*6100* at the right end of the class 1 integron is followed by a partial copy of the same IS. This configuration is seen in In4 of Tn*1696*, but the duplication can be lost by homologous recombination (24, 25). An alignment of RI-a with AbGRI2-0* is shown in Fig. 3B. A deletion generated by the IS*26* on the left (in replicative mode) creates a circular molecule or translocatable unit (TU) (26, 27). This TU was then incorporated into the gene corresponding to ABA1_01225 in A1 (GenBank accession number CP010781) in the *A. baumannii* genome, again likely using the replicative mode to generate the second IS*26* bounding AbGRI2-0* and the 8-bp target site duplication.

### From R1215 to AbaR0.

**FIG 4:**
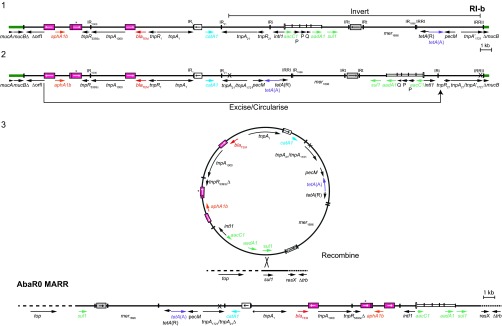
Evolution of the MARR of AbaR0 from RI-b. (A) RI-b configuration, showing the segment inverted by homologous recombination. (B) IS*26*-mediated TU formation. (C) Incorporation of the TU into the GC1 chromosome by homologous recombination. Thick solid green lines indicate adjacent plasmid backbone portions, and black solid and dashed lines represent sequence of the resistance island. The arrows below indicate the extent and orientation of some of the genes and open reading frames, with antibiotic resistance genes shown in color. Insertion sequences are shown as boxes with arrows inside, indicating the orientation of the *tnp* transposase gene. The boxes highlighted in pink, white, and gray represent IS*26*, IS*1*, and IS*6100*, respectively, and the asterisks indicate where the IS*26* is 3 nucleotides different from the standard sequence. Inverted repeats are shown as vertical lines. The *attI1* site of the class 1 integron is shown as a tall open box.

RI-b contains all of the regions of the MARR of AbaR0 but in a different order, as shown in the alignment in Fig. 3C. Multiple steps are required for the evolution from RI-b to AbaR0. First, an inversion between regions of high identity in *tnpA* of Tn*21* and *tnpA* of Tn*1721* creates a hybrid *tnpA* (Fig. 4A). This hybrid is composed of 1,418 bp of Tn*1721* and 401 bp of Tn*21* as present in AbaR0. Second, a different deletion, again generated by the IS*26* on the left in replicative mode and extending to 446 bp into Tn*21* (Fig. 4B), would create a TU. Finally, this TU could then be incorporated into the GC1 *A. baumannii* chromosome, where another *sul1* was already present, through homologous recombination between the two copies of the *sul1* gene (Fig. 4C).

### R1215 is not stably maintained in *A. baumannii*.

*E. coli* containing R1215 was mated with rifampin-resistant *A. baumannii* ATCC 17978 (17978-Rif^r^), using kanamycin, or ampicillin, or kanamycin, ampicillin, spectinomycin, gentamicin, and tetracycline to select for transfer. On two separate occasions, *A. baumannii* resistant to kanamycin (17978-Rif^r^Km^r^) was recovered but was not resistant to the other antibiotics. The *aphA1b* gene was detected by PCR in the 17978-Rif^r^ Km^r^ colonies, accounting for the phenotype, but the IncM replicon was not detected, indicating that R1215 had entered the *A. baumannii* recipient but could not be maintained in it. In R1215, the *aphA1b* gene is located in Tn*6020*, and *A. baumannii* 17978-Rif^r^Km^r^ was screened to determine if the gene was still located in this transposon. Tn*6020* was present, presumably incorporated into the genome, but the location was not determined.

### Related IncM1 plasmids carrying the *bla*_SHV-5_ gene.

**FIG 5:**
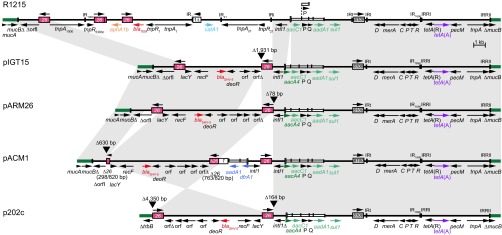
Resistance islands found in the *mucB* gene in the M1 backbone. Thick solid green lines indicate adjacent plasmid backbone portions. Solid horizontal lines represent the sequence of the resistance island, with the extent and orientation of some of the genes and open reading frames indicated by the arrows below. Antibiotic resistance genes are shown in color. Insertion sequences are shown as boxes, with arrows above indicating the orientation of the *tnp* transposase gene and numbers inside indicating the identity of the IS. Inverted repeats are shown as vertical lines. The *attI1* site of the class 1 integron is shown as a tall open box, and each narrow box represents a cassette, with a vertical bar indicating the *attC* site. Gray blocks between lines indicate regions of identical sequence, and black triangles indicate the location of deletions, along with their size.

Some more recent plasmids (listed in Table 1) include a resistance region in the same location as in R1215. The structure of the resistance regions is shown in Fig. 5. In each case, the right-hand end is identical to that in R1215 except for an additional *aacA4* gene cassette in the integron. The left-hand end from the IR of Tn*1721* to the first IS*26* is also present in three of them, but an IS*26*-mediated deletion has removed a short segment in pACM1. The presence of the same Tn*21*-Tn*1721* boundary in this region and in AbaR has been noted (28). In p202c, the left end has been removed together with flanking backbone sequence via an IS*26*-mediated deletion. The *bla*_SHV-5_ gene is in an IS*26*-bounded transposon in each plasmid, though in p202c the internal segment is inverted.

## DISCUSSION

The complexity of the organization of the resistance region of R1215 reflects a complex evolutionary history. The first step is likely to have been the insertion of Tn*1721* into the *mucB* gene. Then, a series of transposition and deletion events occurred within the boundaries of the Tn*1721*, resulting in the loss of the central part of this transposon. In this process, a series of unique boundaries were generated, and these serve to unambiguously link this resistance region to those of the AbaR and AbGRI2 islands in *A. baumannii*. In both cases, an IS*26* formed a TU via an adjacent deletion event (26). This TU was then incorporated into the *A. baumannii* chromosome. The presence of the region found in the AbaR0 MARR that is not in AbGRI2-0*, and vice versa, indicates that both regions were acquired directly from R1215 or a variant of it, rather than one from the other. However, while we obtained evidence that R1215 could transfer into an *A. baumannii* isolate, it does not appear to be stably maintained. Nonetheless, entry would bring the resistance region into the cell and enable the TU formation and transposition or homologous recombination reactions to take place; this occurred in the case of Tn*6020* in the experiments reported here.

Neither the AbaR0 form of the MARR nor the AbGRI2-0* configuration of AbGRI2 has survived unaltered in all members of the GC1 and GC2 complexes, respectively. It is now well established that variant forms are numerous and their formation tends to involve IS*26*-mediated events, particularly deletions occurring adjacent to one end of the IS (13, 15, 16, 19, 20, 29). However, to date, a remnant of the relevant region is present in all reported genomes, indicating that the modern day multiply, extensively, and pan-resistant *A. baumannii* isolates from GC1 and GC2 are all the progeny of a single progenitor for each clone.

## MATERIALS AND METHODS

### Bacterial strains and plasmids.

Plasmid R1215 was obtained from the United Kingdom National Collection of Type Cultures (NCTC 50331), in *Escherichia coli* K-12 strain J53-2 as part of a group of 16 strains containing plasmids of known incompatibility groups for use as controls for PCR-based replicon typing. These strains were screened for resistance to common antibiotics and for the presence of antibiotic resistance genes, mercuric ion resistance genes, class 1 and class 2 integrons, and gene cassettes in class 1 and class 2 integrons, as described elsewhere (22). Strains containing R1215 were maintained under selection with 100 µg/ml ampicillin and 25 µg/ml kanamycin. R1215 was transferred into *E. coli* E294rif^r^ as described previously (7), and transconjugants were recovered on plates containing rifampin (100 µg/ml) to select against the donor and for the recipient and kanamycin to select for R1215. Transconjugant colonies were screened for resistance to antibiotics that the donor strain was resistant to, using L-agar containing the following antibiotics and corresponding concentrations: ampicillin at 100 µg/ml, kanamycin at 20 µg/ml, streptomycin at 25 µg/ml, gentamicin at 8 µg/ml, neomycin at 50 µg/ml, chloramphenicol at 25 µg/ml, tetracycline at 10 µg/ml, and Mueller-Hinton agar containing sulfamethoxazole at 100 µg/ml. Transconjugants were purified and stored at −80°C.

### Plasmid DNA extraction and sequencing.

R1215 plasmid DNA was isolated from a pure transconjugant culture grown overnight at 37°C by using an alkaline lysis miniprep method optimized for the extraction of large plasmids, as previously described (25). The DNA was sequenced at the Australian Genome Research Facility by using a 314 chip on the Ion Torrent PGM platform (Life Technologies). The sequencing reads (191-fold coverage) were assembled *de novo* into contigs by using Geneious version 6.1.6 (Biomatters).

### Plasmid assembly.

The contigs containing backbone sequence overlapped and were assembled with Sequencher 5.1 (Gene Codes). PCR using published primer pairs (17) and RH1581 (5′-GCGGCATATCTGGGTGCTT-3′) in orfI, followed by sequencing of the amplicons, was used to assemble the resistance island. PCR conditions used to detect short amplicons were described previously (30). PCR amplicons were resolved by electrophoresis on 1% (wt/vol) agarose gels with molecular weight standards, stained with ethidium bromide, and visualized using a GelDoc1000 image analysis station (Bio-Rad). Separated PCR products were purified for sequencing by using the QIAquick gel extraction kit (Qiagen Inc., Valencia, CA, USA), following the manufacturer’s protocols. Sequences were assembled using Sequencher, and the Textco (West Lebanon, NH, USA) gene construction kit (version 4.0) was used to draw figures to scale. To confirm the assembly, R1215 plasmid DNA was digested with BsiW1 or BamHI-HF as per the manufacturer’s instructions, and fragments were resolved by electrophoresis on a 0.7% (wt/vol) agarose gel with molecular weight standards, poststained with ethidium bromide, and visualized using a GelDoc100 image analysis station. The observed fragment pattern was compared to digestion patterns predicted from the assembled sequence of R1215 by using the Textco gene construction kit, version 4.0.

### Nucleotide sequence accession number.

The sequence of R1215 has been deposited with GenBank under the accession number KU315015.
